# Prevalence and factors associated with anemia in women of reproductive age across low- and middle-income countries based on national data

**DOI:** 10.1038/s41598-023-46739-z

**Published:** 2023-11-20

**Authors:** Adugnaw Zeleke Alem, Ferry Efendi, Lisa McKenna, Eva Belingon Felipe-Dimog, Dagmawi Chilot, Santo Imanuel Tonapa, Ika Adelia Susanti, Agus Zainuri

**Affiliations:** 1 Department of Epidemiology and Biostatistics, Institute of Public Health, College of Medicine and Health Sciences, University of Gondar, Gondar, Ethiopia; 2 Faculty of Nursing, Universitas Airlangga, Jl. Mulyorejo Kampus C Unair, Surabaya, 60115 Indonesia; 3 School of Nursing and Midwifery, La Trobe University, Melbourne, VIC Australia; 4 Department of Public Health, College of Health Sciences, Kaohsiung Medical University, Kaohsiung City, Taiwan; 5 Nursing Department, Mountain Province State Polytechnic College, Bontoc, Mountain Province Philippines; 6 Department of Human Physiology, School of Medicine, College of Medicine and Health Science, University of Gondar, Gondar, Ethiopia; 7 Center for Innovative Drug Development and Therapeutic Trials for Africa (CDT-Africa), College of Health Sciences, Addis Ababa University, Addis Ababa, Ethiopia; 8 College of Nursing, Kaohsiung Medical University, Kaohsiung, Taiwan; 9 Faculty of Health Science, Universitas Dr. Soebandi, Jember, Indonesia; 10 Faculty of Public Health, Universitas Cenderawasih, Jayapura, Indonesia

**Keywords:** Diseases, Health care, Medical research

## Abstract

Anemia is a global threat among women of reproductive age (WRA), or 15–49 years old women, both in developed and developing countries. Prevalence of anemia in WRA is higher by fourfold in developing countries, based on extensive studies and surveys conducted by WHO and UNICEF. However, there is limited studies that conducted pooled analysis of anemia prevalence in low resource countries. This study aimed to assess the prevalence and factors associated with anemia among women of reproductive age in low- and middle-income countries (LMICs). This study used secondary data from the Demographic and Health Survey (DHS) in 46 low- and middle-income countries during 2010–2021. Descriptive statistics of proportions between pregnant and non-pregnant mothers were assessed. Multilevel binary logistic regression was used to test the factors associated with anemia among women of reproductive age. A total of 881,148 women of childbearing age in LMICs were included. This study found a high prevalence of 45.20% (95% CI 41.21, 49.16) of anemia was observed in among pregnant women and 39.52% (95% CI 33.88, 45.15) anemia was observed in non-pregnant women. Educational status, wealth status, family size, media exposure, and residence were common factors significantly associated with anemia in both pregnant and non-pregnant women. The high global burden of anemia in LMICs continues to underline the need for unusual approaches and target interventions on an individual basis. Global commitment and movement to reduce the prevalence of anemia need to be revisited and redesigned for current circumstances.

## Introduction

Anemia is one of the global health problems faced by people around the world, especially in developing countries as a large contributor^1,2^. The three regions most contributing to anemia worldwide are West Africa, South Asia, and Central Africa^3^. Recent data shows that anemia among women of reproductive age in 82 low and middle-income countries remains a significant challenge^1^. One study noted that the prevalence rate of anemia was 9% in developed countries^4^ while in contrast, in developing countries, the prevalence rate reached 43%, with children and women of reproductive age (WRA) at a greater risk of contracting anemia^5^. The rates of anemia among pregnant women in developed countries such as Australia and the United States of America have been reported at 20% and 18% respectively^6,7^, while in developing countries the number is much higher, for example in Ethiopia where it is 50.1%^8^, Pakistan at 76.7%^9^, and Indonesia at 35.5%^10^. According to the World Health Organization (WHO) report, the global prevalence of anemia in children aged 6–59 months is 39.8% and in WRA or women aged 15–49 years is 29.9%^11^.

Previous research shows that WRA are more likely to get anemia because they lose blood during menstruation and childbirth. This causes the iron (Fe) in their red blood cells to be lost^12^. Anemia can also be caused by pregnancy, specifically because a woman’s body needs more iron (Fe) as the increasing of gestational age^13^. In the socio-economic aspect of WRA, this condition can increase the risk of anemia in WRA, which results in a higher risk of maternal death, prenatal, and perinatal mortality^14^. In general, factors increasing anemia risk for WRA include parity, age, education, knowledge, iron (Fe) consumption, vitamin A deficiency^15^, economic status, nutrition, and diet^1,16^. The World Health Organization study states that the economic level has a role as an underlying factor^17^.

Based on the Demographic and Health Survey, vitamin A deficiency can increase pregnant women’s risk of morbidity and mortality, and anemia caused by iron deficiency can increase the risk of maternal mortality and result in impaired fetal growth^18^. Iron deficiency is the most common and widespread nutritional disorder in developing countries^19^. The risk of anemia during adolescence is higher when women become pregnant. Anemia may also elevate the risk of death among anemic women if excessive bleeding occurs^20^. Iron deficiency, specifically iron deficiency anemia, remains one of the most severe and important nutritional problems in Indonesia. The neonatal household health survey shows the prevalence of anemia being 27% among women aged 15–19 years and 40% among pregnant women. Based on the Nepalese DHS results on anemia, the prevalence of anemia in WRA was 35%. In India, anemia among women is reportedly higher than among men aged 15–49 years with prevalence in women at 57% and men at 25%. However, 26% of WRA are reportedly mildly anemic, and 29% moderately anemic. According to the National Family Health Survey's (NFHS) most recent data, anemia prevalence among women in India has increased from 23% in 2015–2016 to 25% in 2019–2021^21^. These data show that anemia among WRA in developing countries is prevalent. However, none have comprehensively investigated all 46 LMICs between the years 2010 and 2021. It is crucial to emphasize the necessity for regular surveillance of anemia prevalence and its associated factors among WRA to provide insights for stakeholders and guide the development of health intervention strategies. Therefore, the purpose of this study was to examine the prevalence and factors associated with anemia among WRA in low-and middle-income countries.

## Methods

### Study population and data source

This study used the most recent dataset from the Demographic and Health Survey (DHS) conducted in 46 low and middle-income countries (LMICs) from 2010 to 2021. The DHS is a nationally representative, cross-sectional survey in LMICs that provides reliable data on women, men, and children. It uses the same standardized data collection procedures, sampling, questionnaires, and coding, making results comparable across countries. Therefore, all data were appended together to investigate factors associated with anemia in women of reproductive age (15–49 years) in LMICs.

To assure national representativeness, the survey employs a two-stage sampling procedure that involves selection of census enumeration areas from each sampling stratum using a probability proportional to the size of the number of households in each enumeration area in the first stage. In the second stage, households are sampled using systematic random sampling from each enumeration area, which forms the survey clusters. A detailed description of the DHS sampling design and data collection procedures has been found in each country's DHS report. A total of 1,631,752 women of reproductive age were interviewed in 46 LMICs. However, this study was limited to women aged 15–49 years who had been selected for hemoglobin measurement. Finally, this study included a total weighted sample of 881,148 (53,946 pregnant and 827,502 non-pregnant) women of reproductive age.

### Study variables and measurement

The outcome variable for the current study was the anemia status of women. Anemia was measured based on the altitude-adjusted hemoglobin level which was already provided in the DHS data. Anemia in pregnant mothers was operationalized as a categorical variable by predefined cut-off points as not anemic (hemoglobin level ≥ 11 g/dl), mild (hemoglobin level 10–10.9 g/dl), moderate (hemoglobin level 7–9.9 g/dl), and severe (hemoglobin level < 7 g/dl) anemia^22^. Anemia in non-pregnant women was operationalized as a categorical variable by predefined cut-off points as not anemic (hemoglobin level ≥ 12 g/dl), mild (hemoglobin level 10–11.9 g/dl), moderate (hemoglobin level 7–9.9 g/dl), and severe (hemoglobin level < 7 g/dl) anemia^22^. For this analysis, women with mild, moderate, and severe anemia were labeled as anemic because of very small numbers of cases in the category of severe anemia. Hence, pregnant mothers with hemoglobin levels < 11 g/dl and not pregnant with < 12 g/dl were considered anemic and coded as “1” whereas those not anemic were coded as “0”.

Based on previous literature, theoretical and practical significance, we examined independent variables at three levels (individual, household, and community). Level 1 included individual-level factors which were participants’ responses to survey questions on the personal factors. Level 2 factors were characteristics of household from which the individuals came from. The individual level factors included age, educational status of women, marital status, accessing of health care, employment status, termination of pregnancy, smoking status, accessing of health care, mass media exposure and parity for both groups of childbearing age women and contraceptive and body mass index (BMI) for non-pregnant women. Household wealth status, number of household members, sex of household head, source of drinking water, and type of toilet facility were included as household level (level 2) variables. Place of residence was considered as a community-level (level 3) variable.

### Data processing and analysis

Data analysis was carried out using STATA version 16. Descriptive analysis was carried out using frequencies and percentage distributions of the sample for each of the variables. Forest plots were used to visualize the pooled prevalence of anemia among pregnant mothers and non-pregnant women separately. To identify associated factors with anemia, we used multilevel binary logistic regression because DHS data are hierarchical, i.e., individuals are nested within communities. Separate models were fitted for pregnant women, and non-pregnant women since prevalence of anemia were different among the two groups. To cater for the unexplained variability at the community level, we used clusters as random effect. The log of the probability of the anemia was modeled using a three-level model. In particular, four models were constructed. First, we constructed a model containing the outcome variable only (null model) to decompose the total variance into its cluster components. Then model containing only individual-level variables (model I) and model containing individual-level and household level variables (model II) were fitted. Finally, in Model III, we adjusted for all individual, household and community-level variables to estimate anemia and the factors. To select the best-fitted model Log likelihood test was used and the model with the highest Log likelihood was selected (model III). The Intra-class Correlation Coefficient (ICC), and proportional change in variance (PCV) were computed to assess the clustering effect/variability. Intra-class Correlation Coefficient shows the variation in anemia for women of reproductive age due to contextual characteristics and it was computed as follows^23,24^:

$$ICC= \frac{\text{The estimated variance of clusters in each model}}{\text{The estimated variance of clusters in each model }+ 3.29}$$

Proportional change in variance was used to measure total variation attributed to individual and community level variables in the multilevel model (each model) as compared to the null model. We calculated PCV as: PCV % = (VA − VB/VA) × 100; where: VA = variance of the null model, and VB = variance of the model with more factors^24^.

In the initial steps, we conducted bivariable multilevel logistic regression models for each independent variable to choose variables for multivariable multilevel analysis. Variables with a p-value of ≤ 0.20 in the bivariable analysis were selected for the multivariable analysis. The multilevel analyses incorporated a random effect at the cluster level to account for the clustering of individuals within communities. The results of the multivariable analysis were presented as adjusted odds ratios (AOR) along with their 95% confidence intervals (CI) to measure the strength and significance of associations.

### Ethical considerations

This study was conducted in full compliance with the relevant guidelines and regulations governing research in the participating LMICs. All research procedures, including data collection, sampling, and analysis, adhered to the standardized protocols established by the DHS program. The study also obtained ethical approval from ICF International’s Institutional Review Boards (IRBs), ensuring the protection of participants' privacy, confidentiality, and informed consent in accordance with international ethical standards. Detailed information on the ethical standards followed by the DHS program can be found at https://www.dhsprogram.com/methodology/Protecting-the-Privacy-of-DHS-Survey-Respondents.cfm. All methods were performed in accordance with the relevant guidelines and regulations.

## Results

### Background characteristics of study participants

A weighted sample of 881,148 (53,946 pregnant and 827,502 non-pregnant) childbearing-age women in LMICs was included in this study. A larger proportion of pregnant (23,074, 42.8%) and non-pregnant (387,877, 46.9%) women had received secondary education. More than half of pregnant (32,296, 59.9%) and more than two-thirds of non-pregnant (568,567, 68.7%) women used improved drinking water sources. A majority of pregnant (30,587, 56.7%) and non-pregnant (528,353, 63.9%) women had improved toilet facilities. Regarding media exposure, nearly three quarters of pregnant (39,678, 73.6%) and more than three quarters of non-pregnant (660,501, 79.8%) women had media exposure. The majority of study participants, 37,862 (70.2%) pregnant and 520,456 (62.9%) non-pregnant women were rural dwellers (Table 1).

**Table 1:** Frequency distribution of pregnant and non-pregnant study participants in LMICs, 2010–2021.

Variables	Pregnant mothers N = 53,946		Non-pregnant N = 827,502	
	Frequency	Percent	Frequency	Percent
Age (years)				
15–24	26,132	48.5	277,040	33.5
25–34	22,999	42.6	212,413	25.7
35–49	4815	8.9	338,049	40.8
Education status				
Not educated	12,172	22.5	179,000	21.6
Primary	12,031	22.3	149,858	18.1
Secondary	23,074	42.8	387,877	46.9
Higher	6669	12.4	110,766	13.4
Wealth status				
Poorest	12,143	22.5	137,666	16.6
Poorer	11,555	21.4	157,101	19.0
Middle	10,687	19.8	169,909	20.5
Richer	10,764	20.0	179,242	21.7
Richest	8797	16.3	183,584	22.2
Marital status				
Never in union	1874	3.5	254,723	30.8
Married	51,071	94.7	516,937	62.5
Widowed/divorced/ separated	1001	1.8	55,842	6.7
Working status				
Not working	18,288	57.5	189,671	54.1
Working	13,547	42.5	160,942	45.9
Family size				
≤ 5	21,702	40.2	333,401	40.3
5–10	28,227	52.3	451,553	54.6
> 10	4017	7.5	42,548	5.1
Parity				
Nulipara	17,877	33.1	297,605	36.0
Primiparous	14,768	27.4	85,811	10.4
Multiparous	16,494	30.6	364,887	44.1
Grand multiparous	4807	8.9	79,199	9.5
Media exposure				
No	14,252	26.4	166,872	20.2
Yes	39,678	73.6	660,501	79.8
Sex of household head				
Male	43,522	82.5	656,222	79.3
Female	9424	17.5	171,280	20.7
Residence				
Urban	16,084	29.8	307,045	37.1
Rural	37,862	70.2	520,456	62.9
Accessing health care				
Big problem	28,369	52.6	374,454	45.2
Not a big problem	25,576	47.4	452,993	54.8
Termination of pregnancy				
No	45,687	84.7	720,855	87.1
Yes	8258	15.3	106,641	12.9
Health insurance				
No	43,750	83.1	599,590	73.9
Yes	8875	16.9	211,956	26.1
Source of drinking water				
Improved	32,296	59.9	568,567	68.7
Unimproved	21,650	40.1	258,935	31.3
Smoking				
No	52,937	99.5	817,447	99.4
Yes	282	0.5	5,053	0.6
Type of toilet facility				
Improved	30,587	56.7	528,353	63.9
Unimproved	23,359	43.3	299,149	36.1
BMI				
Underweight			127,548	15.6
Normal			458,886	56.2
Overweight/obesity			230,595	28.2
Contraceptive use				
No			456,523	55.2
Yes	–		370,972	44.8

### Prevalence of anemia among pregnant and non-pregnant mothers in LMICs

**Figure 1:**
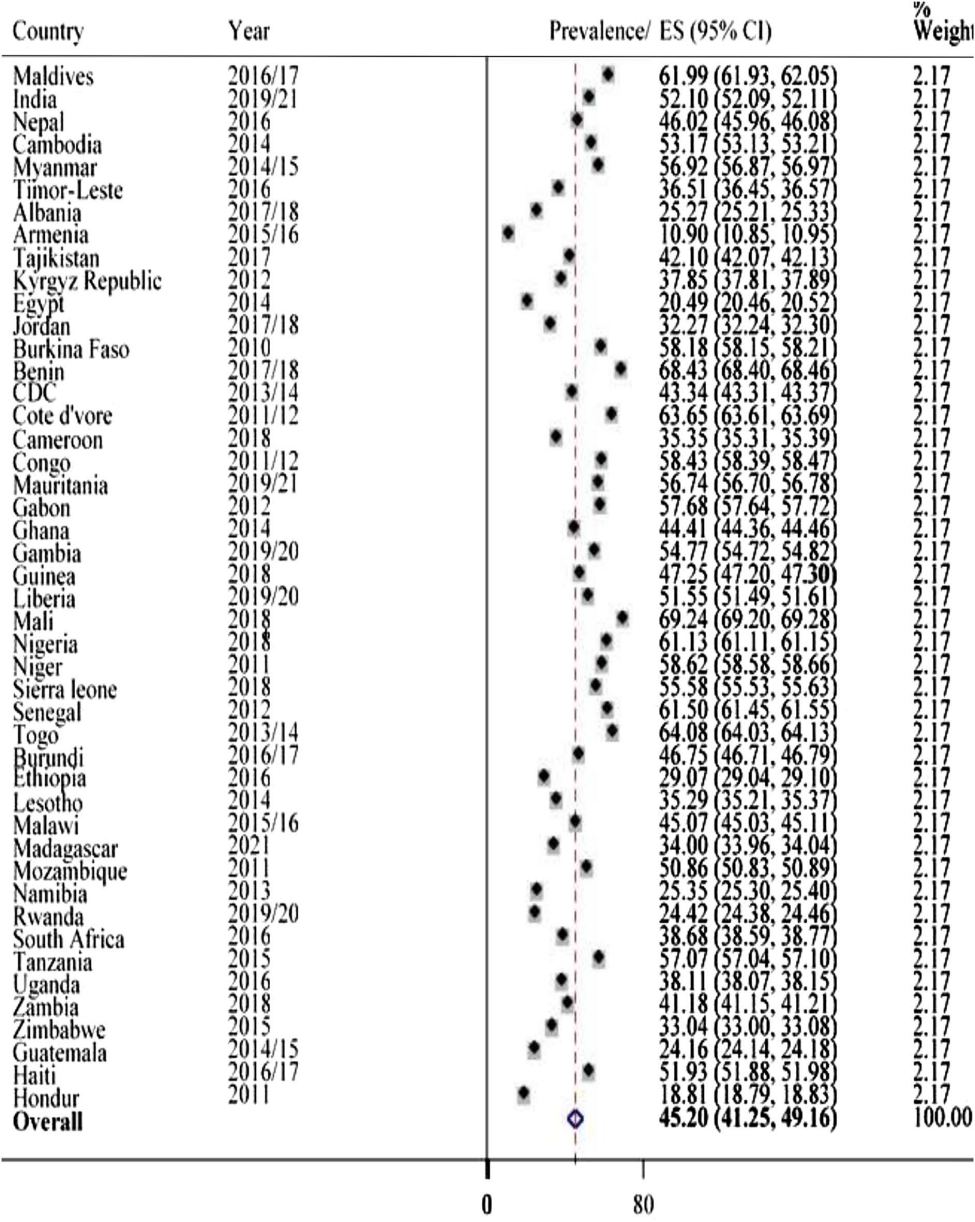
Prevalence of anemia in pregnant mothers in LMICs, 2010–2021.

**Figure 2:**
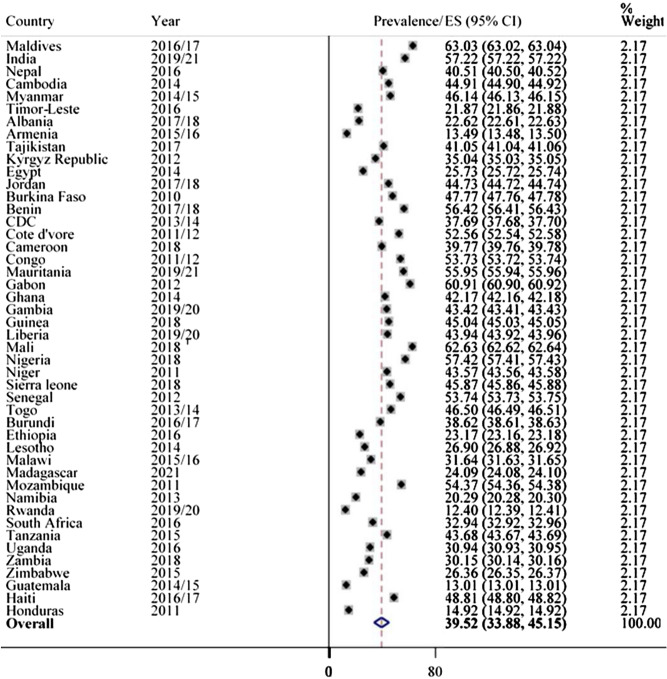
Prevalence of anemia in non-pregnant mothers in LMICs, 2010–2021.

The study showed that a high prevalence of 45.20% (95% CI 41.25, 49.16) of anemia was observed in LMICs among pregnant women, ranging from 10.90% in Armenia to 69.24% in Mali (Fig. 1). The prevalence of anemia in non-pregnant women was 39.52% (95% CI 33.88, 45.15), ranging from a low of 12.40% in Rwanda to a high of 63.03% in Maldives (Fig. 2). In the analysis, Mali has consistently had higher anemia prevalence in both pregnant (69.24%) and non-pregnant (62.63%) women.

### Multilevel analysis

#### Random parameters and model selection

The random effect analysis in the null model was used to examine the cluster effect on anemia among pregnant and non-pregnant mothers. The results implied variability in anemia across the clusters among pregnant (ICC = 1.99%) and non-pregnant mothers (ICC = 13.87%) which indicates that the cluster accounted for a 1.99% and 13.87% variance in anemia among pregnant and non-pregnant mothers, respectively. In Model III, the variability in anemia decreased across clusters among pregnant and non-pregnant mothers. In Model I, Model II, and Model III, the explained variance among pregnant mothers was 2.98%, 10.45% and 14.92%, respectively. This implied that a large amount of variances in anemia has been explained by model III. Regarding model comparison, we used likelihood ratio to assess model fitness. Consequently, to identify factors associated with anemia, model III was selected as the best fitted model due to the large likelihood ratio (p-value < 0.001) (Table 2).

**Table 2:** Random effect parameters and model comparison.

	Cluster level variance	ICC (%)	PCV (%)	Log likelihood	Assumption (chi^2^(df), p value))
Models for pregnant mothers					
Null model	0.067	1.99	Reference	− 36,766.157	Null model is nested in Model I (LR chi2(9) = 720.08, < 0.001)
Model I	0.065	1.94	2.98	− 36,406.115	Model I is nested in Model II (LR chi2(10) = 204.09, ≤ 0.001)
Model II	0.058	1.73	10.45	− 36,304.071	
Model III	0.057	1.70	14.92	− 36,298.683	Model II is nested in Model III (LR chi2(1) = 10.78, 0.001)
Models for non-pregnant mothers					
Null model	0.530	13.87	Reference	− 533,346.2	Null model is nested in Model I (LR chi2(10) = 2577.88, < 0.001)
Model I	0.523	13.72	1.32	− 532,057.26	Model I is nested in Model II (LR chi2 (10) = 1394.50, ≤ 0.001)
Model II	0.517	13.58	2.45	− 531,360.01	
Model III	0.515	13.53	2.83	− 531,314.53	Model II is nested in Model III (LR chi2(1) = 90.95, ≤ 0.001)

*ICC* Intra-class Correlation Coefficient, *PCV* Proportional Change in Variance.

#### Factors associated with anemia among pregnant mothers

We found that educational status, wealth status, family size, media exposure, and residence were common factors significantly associated with anemia in pregnant and non-pregnant women. While termination of pregnancy, contraceptive use, parity, and sources of drinking water were factors significantly associated with anemia in non-pregnant women. Besides these, age and health insurance were factors significantly associated with anemia in pregnant mothers.

The odds of anemia among pregnant were 1.75 (AOR = 1.75; 95% CI 1.63, 1.89) for mothers with no formal education, 1.19 (AOR = 1.19; 95% CI 1.11, 1.28) for primary education and 1.29 (AOR = 1.29; 95% CI 1.21, 1.37) for secondary education as compared to those with higher education. Similarly, the odds of anemia among non-pregnant were 1.23 (AOR = 1.23; 95% CI 1.20, 1.25) for mothers with no formal education and 1.12 (AOR = 1.12; 95% CI 1.11, 1.14) for secondary education as compared to those with higher education. Likewise, increased odds of anemia among pregnant (AOR = 1.26; 95% CI 1.17, 1.35) and non-pregnant (AOR = 1.24; 95% CI 1.21, 1.27) women were observed from > 10 family member households.

The odds of anemia among pregnant women were 23% (AOR = 0.77; 95% CI 0.72, 0.82) lower among ≥ 35 years as compared to women aged 15–24 years. The odds of anemia among pregnant women was 1.08 (AOR = 1.08; 95% CI 1.04, 1.13) times higher in those not exposed to mass media exposure than those who were exposed. Compared to pregnant women with richest wealth status, women of poorest (AOR = 1.32; 95% CI 1.23, 1.41), poorer (AOR = 1.17; 95% CI 1.09, 1.25), middle (AOR = 1.10; 95% CI 1.03, 1.18), and richer (AOR = 1.12; 95% CI 1.05, 1.19) wealth status have the increased risk for anemia. Pregnant women without health insurance coverage were 1.07 (AOR = 1.07; 95% CI 1.01, 1.12) times more likely to have anemia that those with health insurance. Lastly, pregnant women from rural areas were 1.08 (AOR = 1.08; 95% CI 1.03, 1.13) times higher to have anemia than those living in urban areas.

Non-pregnant women from poorest, poorer, middle and richer households had 1.23 (AOR = 1.23; 95% CI 1.19, 1.24), 1.13 (AOR = 1.13; 95% CI 1.10, 1.15), 1.11 (AOR = 1.11; 95% CI 1.09, 1.26), and 1.06 (AOR = 1.06; 95% CI 1.04, 1.07) times higher odds of having anemia as compared to women from richest households. Moreover, odds of anemia among non-pregnant women were higher in those living in rural areas (AOR = 1.08; 95% CI 1.06, 1.09), not exposed to mass media (AOR = 1.09; 95% CI 1.06, 1.10), not using contraceptive methods (AOR = 1.15; 95% CI 1.14, 1.17), with unimproved drinking water source (AOR = 1.22; 95% CI 1.20, 1.25) and ever having termination of pregnancy (AOR = 1.06; 95% CI 1.05, 1.08). Among non-pregnant and lactating women, the odds of anemia were 11% (AOR = 0.89; 95% CI 0.84, 0.95) lower among smokers than with non-smokers (Table 3).

**Table 3:** Multi-level binary logistic regression of factors associated with anemia among pregnant and non-pregnant women in LMICs, DHS 2010–2021.

Variables	Category	Pregnant mothers	Non-pregnant women
		AOR (95% CI)	AOR (95% CI)
Age (years)	15–24	1	1
	25–34	0.88 (0.87, 0.97)**	0.97 (0.95, 1.01)
	35–49	0.77 (0.72, 0.82)**	0.98 (0.97, 1.03)
Education status	Not educated	1.75 (1.63, 1.89)**	1.23 (1.20, 1.25)**
	Primary	1.19 (1.11, 1.28)**	0.99 (0.98, 1.02)
	Secondary	1.29 (1.21, 1.37)**	1.12 (1.11, 1.14)**
	Higher	1	1
Wealth status	Poorest	1.32 (1.23, 1.41)**	1.23 (1.19, 1.24)**
	Poorer	1.17 (1.09, 1.25)**	1.13 (1.10, 1.15)**
	Middle	1.10 (1.03, 1.18)**	1.11 (1.09, 1.26)**
	Richer	1.12 (1.05, 1.19)**	1.06 (1.04, 1.07)**
	Richest	1	1
Family size	≤ 5	1	1
	5–10	1.05 (1.06, 1.15)**	1.07 (1.06, 1.08)**
	> 10	1.26 (1.17, 1.35)**	1.24 (1.21, 1.27)**
Media exposure	Yes	1	1
	No	1.08 (1.04, 1.13)**	1.09 (1.06, 1.10)**
Sex of household head	Male	1	1
	Female	1.06 (0.99, 1.11)	0.98 (0.97, 1.02)
Residence	Urban	1	1
	Rural	1.08 (1.03, 1.13)*	1.08 (1.06, 1.09)*
Accessing health care	Not big problem	1	1
	Big problem	1.01 (0.98, 1.05)	1.01 (0.99, 1.02)
Termination of pregnancy	No	1	1
	Yes	1.03 (0.98, 1.05)	1.06 (1.05, 1.08)*
Covered by health insurance	Yes	1	1
	No	1.07 (1.01, 1.12)*	1.02 (0.98, 1.04)
Type of source of drinking water	Improved	1	1
	Unimproved	0.97 (0.93, 1.01)	1.22 (1.20, 1.25)**
Smoking	No	1	1
	Yes	0.85 (0.67, 1.08)	0.89 (0.84, 0.95)**
Type of toilet facility	Improved	1	1
	Unimproved	1.02 (0.98, 1.05)	0.99 (0.97, 1.01)
Contraceptive use	Yes		1
	No		1.15 (1.14, 1.17)**
Parity	Nulliparous		1
	Primiparous		0.99 (0.97, 1.01)
	Multiparous		1.07 (1.05, 1.09)**
	Grand multiparous		1.07 (1.04, 1.09)**

**p-value < 0.001, *p-value < 0.05.

## Discussion

To the best of our knowledge, this study is the first to systematically determine the prevalence and associated factors of anemia between reproductively aged pregnant and non-pregnant women across LMICs. Our analysis revealed that the prevalence of anemia among pregnant (45.2%) and non-pregnant (39.5%) women in the 46 LMICs were higher compared to the global prevalence in 2019 (36% among pregnant women, and 30% among non-pregnant women)^25^. Based on targets of the World Health Organization (WHO), the prevalence of anemia among reproductive-aged women should decrease to ≤ 15.2% by 2025^26^. Our findings found that the prevalence of anemia among reproductive-aged women in LMICs, both pregnant and non-pregnant women, remains high and far from the global target, comparable with previous studies in^1,16,27^.

Among the 46 LMICs, Mali has consistently higher anemia prevalence in both pregnant (69%) and non-pregnant (63%) women, while the lowest was among pregnant women in Armenia (11%) and non-pregnant women in Rwanda (12%). Previous studies have suggested that, in Mali, vaginal and parasitic infection, and sociocultural imposed food restrictions contributed to increased rates of anemia among pregnant women^28,29^. Therefore, prevention and control of vaginal and parasitic infection, and health information regarding nutritional food during pregnancy might help in mitigating anemia in Mali and neighboring countries with similar public health challenges.

Further, we found that, between pregnant and non-pregnant women in LMIC, pooled anemia prevalence was higher among pregnant than among non-pregnant women, similar to some studies in China^30^, and in Karachi, Pakistan^31^. The higher rate of anemia in pregnant women is due to the fact that, during pregnancy, iron requirement is higher due to greater plasma volume expansion to support the pregnancy, leading to decreased hemoglobin levels that increase the risk of pregnant women developing anemia^32,33^. This emphasizes the importance of antenatal iron supplementation for preventing anemia^34,35^ and decreasing the risk of pregnant women having anemia^36–38^. The WHO recommends the daily oral intake of supplements containing iron should be 30–60 mg and folic acid at 0.4 mg throughout pregnancy among women for prevention of anemia, as well as prevention of puerperal infection, low birth weight, and premature birth^39^.

In this study, we found that education and wealth status of pregnant and non-pregnant women were associated with anemia. Women who were not educated and had secondary level education were more likely to have anemia compared to women with higher education. Similarly, women from the poorest to richer wealth classes were more likely to have anemia compared to the richest group of women. These findings are comparable to previous studies in LMICs including Nepal^40^, Ethiopia^41^, India^42^, Nigeria^43^, and Pakistan^44^ linking socioeconomic factors with anemia. Education has a strong relationship with income and wealth. Individuals with better education tend to earn more income, and finishing at least a college degree equates to a better salary^45^. In addition, women with better education probably have better knowledge and health behavior which in turn encourages them to adopt healthier lifestyles such as eating nutritious food, better health decision-making, and better hygienic habits^41^. On the other hand, women with higher wealth status may have better financial capacity to access material goods and services which include healthcare services^46^. These situations potentially result in better access to nutritious food and better healthcare services that contribute to prevention and control of anemia in women with higher education and the richest wealth status, as compared to women with lower education and wealth status. Therefore, anemia control programs, such as free food supplements and nutrition counseling, among women of lower education and marginalized poor women in LMICs potentially help in reducing anemia prevalence.

We found in this study that a family size of five or more members increased the likelihood of LMIC pregnant and non-pregnant women having anemia compared to women with < 5 family members. This finding is similar to studies in Ethiopia^47^ and Pakistan^44^ wherein women with five or more family members had a two-fold increased risk of anemia. This may be a result of food poverty and reduced nutrient consumption that could have averted anemia due to bigger families^47^. It has been noted that, as household size increases, the food insecurity rate increases^48^. The availability of affordable nutritious food containing iron for women with large family sizes might be a strategy to mitigate anemia in food-insecure communities.

Women from LMICs who were not exposed to mass media were more likely to have anemia. This finding is similar to results of a study in Nepal wherein women who were exposed to newspapers and radio were less likely to have anemia^49^. Another similar experimental study in Jordan found that 54% of women from the intervention group who were exposed to health information through video presentation combined with a PowerPoint presentation on anemia in pregnancy were non-anemic; while only 24% of the control group of women who received standard antenatal care including iron supplements only were non-anemic^50^. These studies indicate the vital role of mass media in promotion of health and prevention of diseases. It was suggested that exposing women to health information via health education programs is effective for educating them on the various information regarding a proper diet, which may help them become familiar with food classifications and improve their ability to choose foods that increase hemoglobin levels, such as those high in iron, protein, and vitamin C^50^. Similar to this, a quasi-experimental study conducted in Indonesia revealed that pregnant women benefited from receiving health education and counseling through visual handbooks by having increased hemoglobin and hematocrit levels^51^. Currently, mass media channels such as information brochures, posters, press and article advertisements, television, radio communications, and social media (e.g. Twitter, Facebook, and WhatsApp) are now being promoted to spread awareness of public health issues^52^. Exposing people to various media channels that provide information on the promotion of health and prevention of diseases may be a crucial technique to lower anemia prevalence among reproductive-aged women in LMICs.

We found that rural women were also more likely to have anemia compared with urban women in LMICs. A similar result has been reported in previous studies from LMICs including in Pakistan^44^, Ethiopia^47^, and Nigeria^53^. Anemia may be more common in rural locations due to high rates of poverty, and inadequate access to healthy food and safe water, education, and basic medical services. This situation can also explain the increased likelihood of non-pregnant women with unimproved sources of drinking water having anemia. Poor drinking water source is a common problem in rural poor communities that resulted in several public health issues^54^ such as anemia. Previous investigations^55,56^ observed that unavailability and poor sources of water supply were found to be associated with anemia among reproductive-aged women in developing countries. Exposure to poor-quality water supply causes spread of parasitic infections such as malaria and schistosomiasis^57^ that are linked to prevalence of anemia^58–60^. It is commonly known that schistosomiasis is a parasitic infection that causes persistent blood loss, which results in anemia^61,62^. A study in Chile also found that drinking water contaminated with arsenic increased the risk of anemia in women^63^. Arsenic is a chemical that is found in the groundwater system at a higher concentration, especially in shallow tube wells^64^. Groundwater sources such as wells are commonly used as household water supplies in LMICs^65^. Therefore, deworming in areas where parasitic infection is common, iron supplementation, and intervention in safe water sources and sanitation systems all play a crucial part in minimizing anemia and other nutritional losses brought about by water-borne infections^66^.

In our study, we found that pregnant women aged 25–34 and 35–49 years had decreased risk of having anemia by 12% and 23%, respectively, as compared with those in the 15–24-year age bracket, comparable to a study conducted in Nepal^49^. The reduced risk of anemia among pregnant women aged 25 years and up might be due to better health decision-making in taking good care during pregnancy as compared to younger pregnant women. For instance, several studies in LMICs have shown that ≥ 25-year-old women were more compliant in taking prescribed antenatal iron supplements than women < 25 years of age^67,68^. Iron supplementation is part of the antenatal care package for the treatment and prevention of anemia during pregnancy^69^.

In LMIC, non-pregnant women who were not using contraceptives were more likely to have anemia compared to women who were using them. This result was similar to previous studies in that contraceptive use lowered the risk of women having anemia^70,71^. In Nepal, it was found that women using hormonal injectable contraceptives (Depo-Provera) had higher levels of hemoglobin concentration^72^. A strong negative relationship between hormonal contraceptive use and anemia was discovered^70,73^, supporting the findings of this study and pointing to a potential preventive effect of hormonal contraception against anemia. Reduced menstrual flow as a result of hormonal contraceptive use is the likely primary mechanism^74^. Promoting the use of modern hormonal contraceptive methods among sexually active women has the potential to decrease the prevalence of anemia among non-pregnant women in LMICs.

Health insurance combines risks and assets from a group of people to protect each person from the financial burden of paying medical bills brought on by sickness, accident, or disability^75^. It could be an effective substitute for out-of-pocket expenses and their associated financial costs^76^. In this study, non-pregnant women not covered by health insurance were more likely to have anemia, which inversely suggests that women with health insurance have decreased risk of having anemia. There is limited literature investigating the connection between health insurance and anemia among women. Nonetheless, a longitudinal household survey in Burkina Faso, West Africa, found that households that had community health insurance membership were 2.23 times more likely to utilize healthcare services compared to those without^77^. It is possible that health-insured women from LMICs used every healthcare service related to the promotion of nutrition such as iron supplementation for the treatment and prevention of anemia which reduced their risk of having anemia. Therefore, the inclusion of all reproductive-aged women in health insurance coverage potentially reduces anemia prevalence in LMICs.

Non-pregnant women with pregnancy termination history, also known as abortion, were found to be more likely to have anemia than women who did not. Similarly, a study in Trinidad and Tobago, an island country in the Caribbean, found that women with 2–3 terminated pregnancies were more likely to have anemia than those who did not have an abortion^78^. The increased likelihood of women with a history of abortion having anemia may be linked to hemorrhage, one of the complications of abortion, resulting in an increased risk of anemia^79^. Post-abortion hemorrhage might result from blood loss due to cervical or vaginal laceration, and uterine atony brought about by termination of pregnancy^79^. To address the complications of pregnancy termination, such as hemorrhage, assessment of women's hemodynamic status and anticipation of possible blood transfusion are recommended^80^.

In this study, multipara and grand multipara women were 1.07 times more likely to have anemia than nullipara women (no childbirth history). This finding is comparable to other investigations in developing countries^81,82^. Several studies also show similar results in the relationship between high parity and prevalence of anemia. In Brazil, women with more than two children had a twofold risk tof having anemia compared to women with < 2 children^83^. In Nepal, women with > 4 children were more likely to have anemia in 2016, and where the prevalence of anemia, in fact, increased between 2006 and 2016^84^. On the contrary, a study in China found that women with more than one child had a decreased risk of having anemia in rural areas of Western China in 2001 and 2005^85^. The family planning policy of China at the time, allowing more than one child based on sub-population features, such as socioeconomic situations, may be one explanation for this contradicting conclusion^85^. This indicates that women of higher socioeconomic class who choose to have more than one child may have a lower risk of anemia than women of low socioeconomic condition. For women from households of poor socioeconomic status, planning the number of children that the family can sustain and support may be a way to reduce the risk of women having anemia. As shown in an Indian regression decomposition study, a decline in the proportion of children under the age of five was responsible for a 6% hemoglobin concentration increase in pregnant women between 2006 and 2016^86^.

The fact that this study used combined data from 46 nationally representative DHS surveys in LMICs was one of its main advantages. This high sample size, therefore, has sufficient ability to identify the actual impact of the independent factors. To obtain accurate estimates and standard errors, the sample weight was also used throughout the analysis. Due to the cross-sectional nature of the present study, it is not possible to establish causal links between anemia and determined predictors. Additionally, because this study utilized publicly available secondary data, co-equally relevant factors associated with anemia such as dietary patterns, parasite infections including hookworm, hospital admission history, and use of food supplements including iron and folic acid, were excluded.

## Conclusion

Anemia in reproductive-aged women is high in LMICs, especially among pregnant women. Out of the 46 LMICs examined, only Armenia among both groups (pregnant, and non-pregnant women), Rwanda among non-pregnant women, and Honduras among non-pregnant women were able to meet the 2025 global target of having ≤ 15% anemia prevalence. The identification of LMICs with high anemia prevalence provides an unparalleled opportunity for global and country leaders, policy-makers, and program managers to revisit strategies, reformulate policies and reallocate resources targeting communities at risk.

It is essential to promote dietary variety and consumption of iron-rich foods for all reproductive-aged women. To compensate for menstrual iron losses and to meet pregnancy iron requirements, daily iron supplements are advised especially for younger pregnant women aged 15–24 years. In addition to the aforementioned nutrition-specific interventions, nutrition-sensitive interventions such as raising women's educational levels, promoting better reproductive health practices, such as having the appropriate number of children that a family can support and sustain, ensuring access to high-quality maternal health care, disseminating information about prevention and treatment of anemia through various mass media channels, and improving sanitation and access to safe water supply, continue to be crucial. Additionally, the fight against anemia and other health indicators among women in LMICs can be significantly improved by providing women with sustainable economic opportunities. The results of the present study can provide a baseline reference for international organizations for future investigation of anemia in women from LMICs.

However, there are significant challenges due to economic disparities and cultural misunderstandings. To address these, a comprehensive approach is needed, including economic empowerment through training and finance, culturally sensitive awareness campaigns, better healthcare systems with widespread anemia screening, personalized education in local languages using technology, focused policies, and strategies suited to different cultures. By working together to overcome these barriers and using tailored solutions, the global community can make real progress in reducing anemia among women in LMICs.

## Data Availability

The datasets generated and/or analyzed during the current study are available in the [DHS] repository, https://dhsprogram.com/data/available-datasets.cfm.
